# A specific family of interspersed repeats (SINEs) facilitates meiotic synapsis in mammals

**DOI:** 10.1186/1755-8166-6-1

**Published:** 2013-01-01

**Authors:** Matthew E Johnson, Ross A Rowsey, Sofia Shirley, Catherine VandeVoort, Jeffrey Bailey, Terry Hassold

**Affiliations:** 1 Washington State University, School of Molecular Biosciences and Center for Reproductive Biology, Biotechnology-Life Science Building, 1715 NE Fairway Road, Pullman, WA 99164, USA; 2 California National Primate Research Center, University of California, Davis, Davis, CA 95616, USA; 3 University of Massachusetts Program in Bioinformatics and Integrative Biology and Division of Transfusion Medicine, 55 Lake Avenue N, Worcester, MA 01605, USA

**Keywords:** Meiosis, Synaptonemal complex, Chromatin Immunoprecipitation (ChIP), SINE, Synapsis, SYCP3, Mouse, Macaque

## Abstract

**Background:**

Errors during meiosis that affect synapsis and recombination between homologous chromosomes contribute to aneuploidy and infertility in humans. Despite the clinical relevance of these defects, we know very little about the mechanisms by which homologous chromosomes interact with one another during mammalian meiotic prophase. Further, we remain ignorant of the way in which chromosomal DNA complexes with the meiosis-specific structure that tethers homologs, the synaptonemal complex (SC), and whether specific DNA elements are necessary for this interaction.

**Results:**

In the present study we utilized chromatin immunoprecipitation (ChIP) and DNA sequencing to demonstrate that the axial elements of the mammalian SC are markedly enriched for a specific family of interspersed repeats, short interspersed elements (SINEs). Further, we refine the role of the repeats to specific sub-families of SINEs, B1 in mouse and AluY in old world monkey (*Macaca mulatta*).

**Conclusions:**

Because B1 and AluY elements are the most actively retrotransposing SINEs in mice and rhesus monkeys, respectively, our observations imply that they may serve a dual function in axial element binding; i.e., as the anchoring point for the SC but possibly also as a suppressor/regulator of retrotransposition.

## Background

The formation of haploid gametes is dependent on several major processes that occur during meiotic prophase; i.e. following genome replication homologs must pair, synapse, and exchange material in advance of segregation at anaphase I [1-5]. These processes are aided by the formation of the synaptonemal complex, a tripartite structure consisting of two axial elements (AEs) and a transverse filament that brings the AEs into close alignment [6-8]. Chromosomal DNA is thought to bind to the SC via the AE protein SYCP3 [7,9,10]. However, previous attempts to identify mammalian DNA sequences that seed this interaction involved limited sequencing efforts [11,12] thus; it is not yet clear whether specific DNA sequences are necessary for formation of the SC.

## Results

### Mouse ChIP

We assessed this interaction in male mice, utilizing ChIP followed by DNA sequencing to determine which, if any, DNA elements bind with SYCP3 (either directly or in a protein complex that includes SYCP3) during the formation of the SC. Specifically, we isolated seminiferous tubules from the testes of C57BL/6J male mice and pulled down the protein-DNA complex using an antibody against SYCP3. Due to the small amount of starting testis material, DNA yields were limited (0.5-2 ng/ul). Thus, we first employed whole genome amplification (WGA) of DNA from test and mock pull-downs to generate sufficient DNA for subsequent subcloning efforts. Subclones were sequenced by ABI sequencing and aligned against the genome assembly (NCBI37/mm9, July 2007); we only accepted clones with at least 98% or better sequence identity to the mouse genome assembly [13].

**Figure 1:**
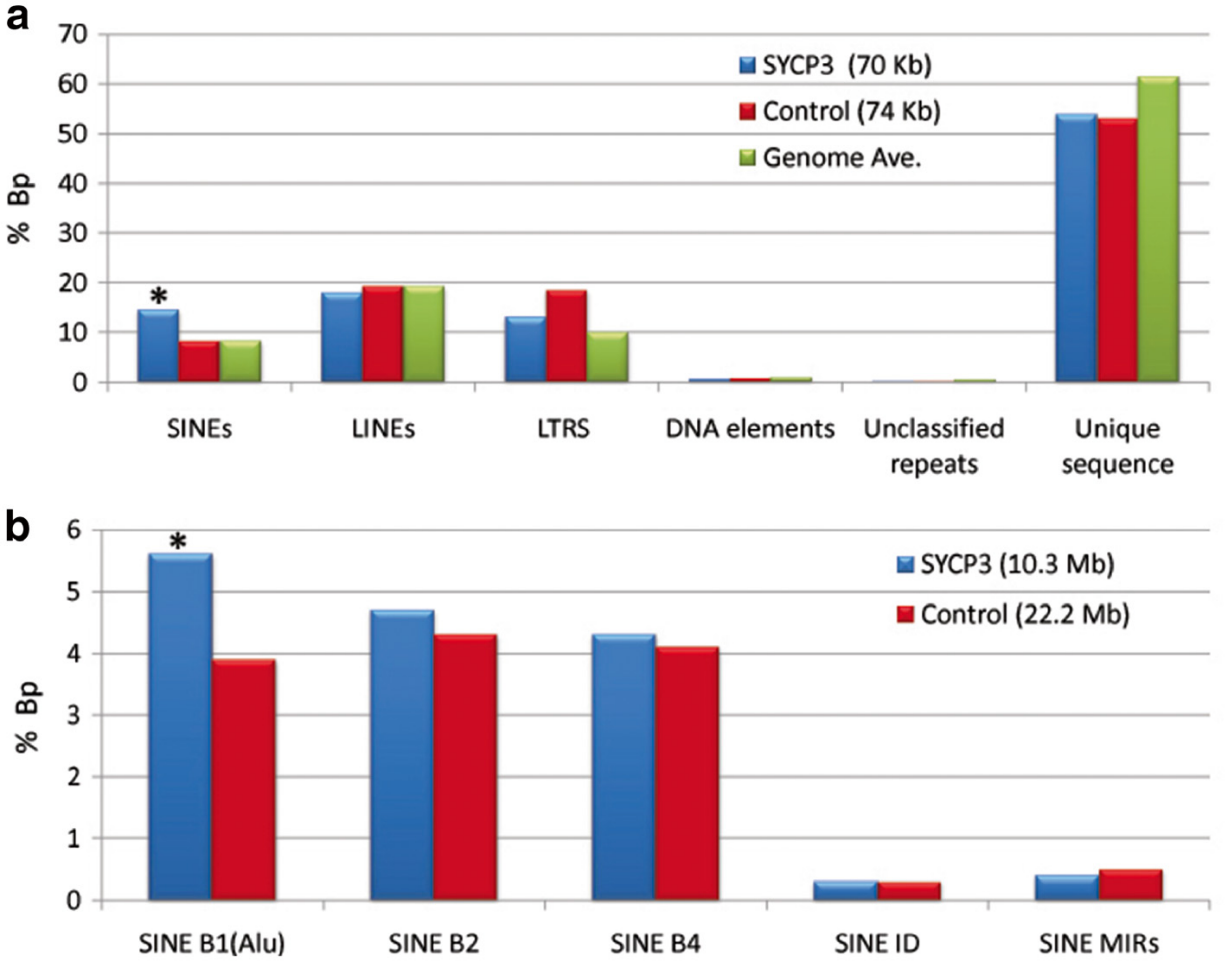
**Sequence analysis from C57BL/6J male mice.** Sequence analysis of DNA from seminiferous tubules of C57BL/6J male mice, amplified following test (SYCP3) or control (no antibody) ChIP pull-downs. Results are expressed as the proportion of base pairs observed/overall number of base pairs sequenced for: (**a**) each of six categories of DNA, based on sequence analyses of subcloned and (**b**) each of five categories of mouse SINE elements, based on 454 genome sequencing. Asterisks indicate significant differences between test and control groups at p<0.0001; data are also provided in Tables 1 and 2.

In total, we analyzed 70.9 Kb from 239 test (anti-SYCP3 ChIP) subclones and 55.3 Kb from 180 control (mock pull-down) subclones (Table 1; Figure 1a). The types of sequence features contained within the test and control sequences were compared by placement against the mouse genome assembly and RepeatMasker analysis [13,14]. Only one category of DNA, the SINE interspersed repeat element, was over-represented in the test group, accounting for 13.4% of bp in the test group but only 9.2% of bp in the control group (p<0.0001; Figure 1a). Thus, these results suggest a preference for SINEs in the formation of the AEs but, because of the relatively limited amount of sequence data, we were unable to determine whether specific sub-families of SINEs were responsible for this association.

**Table 1:** Distribution of different categories of DNA, based on sequence analysis of subclones from test (SYCP3) and control (no antibody) ChIP experiments of male

**DNA type**	**Test no. of base pairs (%)**	**Control no. of base pairs (%)**	**Mouse genome ave. (%)**
**SINEs**	9483 (13.4)	5065 (9.2)	8.2
**LINEs**	13023 (18.3)	10832 (19.6)	19.2
**LTRs**	10422 (14.7)	9959 (18.0)	9.9
**DNA Elements**	533 (0.8)	360 (0.6)	0.9
**Unclassified/other repeats**	205 (0.3)	58 (0.1)	0.4
**Unique sequence**	37325 (52.6)	29114 (52.6)	61.4
**Total Bp Sequenced**	70991 (100.1)	55388 (100.1)	100.0

### Mouse ChIP-Seq

Accordingly, we conducted a second set of experiments to determine which, if any, of the five mouse SINE family members -- B1, B2, B3, B4/RSINE, ID and MIR/MIR3 – might specifically interact with SYCP3. For this analysis, we utilized the Roche 454 GS FLX pyrosequencing platform, enabling us to generate a much larger data set. In total, we analyzed 10.3 Mb of test sequence and 22.2 Mb of control sequence reads (Table 2), and confirmed the major finding from the previous subcloning experiment; SINEs were over-represented in the test group (15.2% of bp) by comparison with the control group (13.1% of bp). Analyses of the individual SINE families demonstrated that the effect was attributable to B1 elements (Figure 1b). Taken together, these studies indicate that one type of interspersed repeat element, B1 SINEs, are preferentially utilized as binding sites in the formation of the axial elements of the synaptonemal complex.

**Table 2:** Distribution of different categories of DNA based on 454 GS FLX Titanium sequencing from test (SYCP3) and control (no antibody) ChIP experiments of male mice

**DNA type**	**Test no. of base pairs (%)**	**Control no. of base pairs (%)**	**Mouse genome ave. (%)**
**SINEs, Total**	1571293 (15.3)	2896767 (13.1)	8.2
**SINE: Alu/B1**	578758 (5.6)	856113 (3.9)	2.7
**SINE: B2**	481108 (4.7)	958107 (4.3)	2.4
**SINE: B4**	442437 (4.3)	908997 (4.1)	2.4
**SINE: ID**	27144 (0.3)	55704 (0.3)	0.3
**SINE: MIR**	41623 (0.4)	116746 (0.5)	0.6
**LINEs**	2022182 (19.6)	3930338 (17.7)	19.2
**LTRs**	1354824 (13.2)	2820553 (12.7)	9.9
**DNA Elements**	85639 (0.8)	221236 (1.0)	0.9
**Unclassified/other repeats**	44615 (0.4)	97630 (0.4)	0.4
**Unique sequence**	5220735 (50.7)	12236884 (55.1)	61.4
**Total Bp Sequenced**	10299288 (100.0)	22203408 (100.0)	100.0

### Macaque ChIP

**Figure 2:**
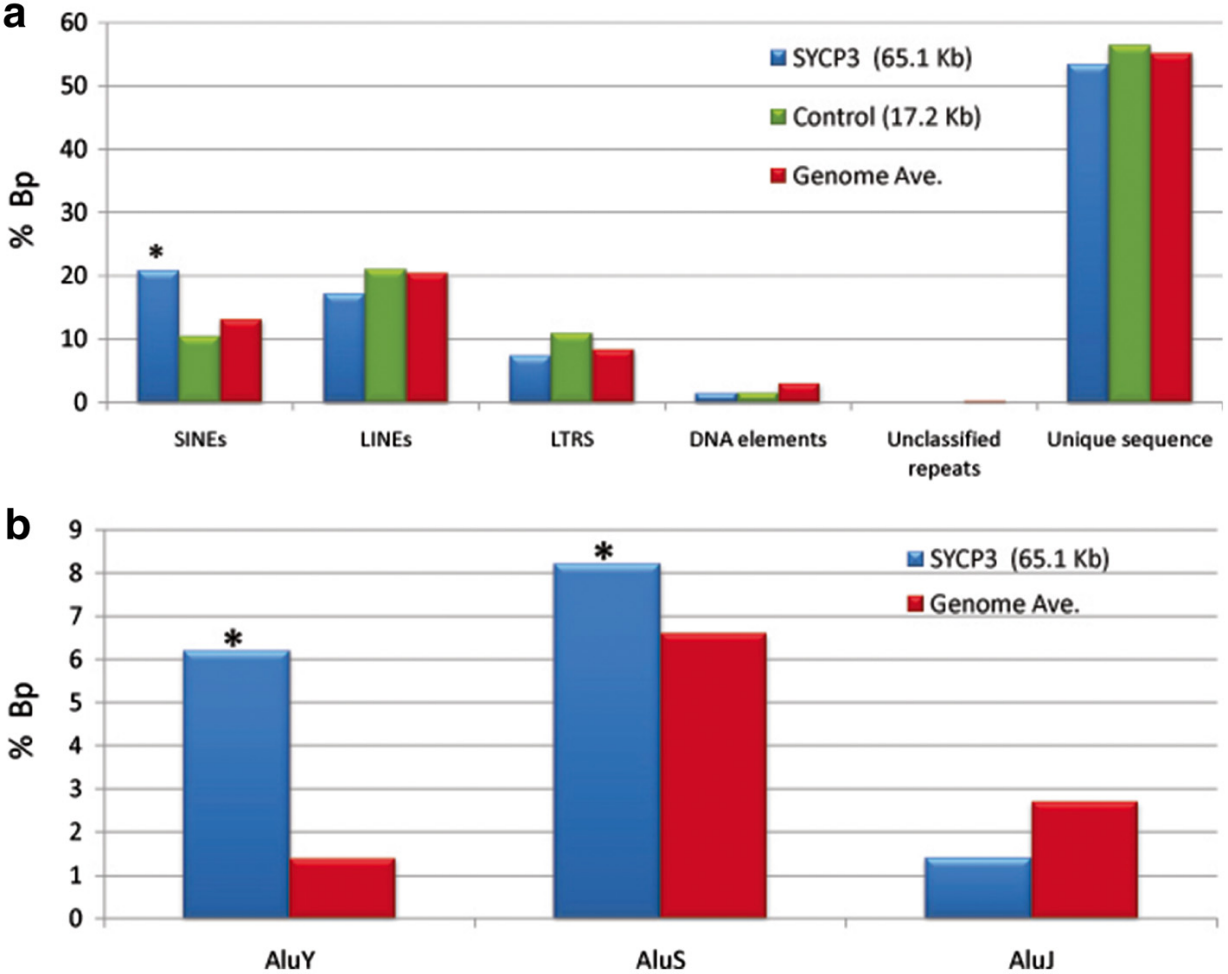
**Sequence analysis from rhesus macaque males.** Sequence analysis of DNA from seminiferous tubules of rhesus macaque males, amplified following test (SYCP3) or control (no antibody) ChIP pull-downs. Results are expressed as the proportion of base pairs observed/overall number of base pairs following 454 genome sequencing for: (**a**) each of six categories of DNA and (**b**) each of three categories of Alu elements. Asterisks indicate significant differences between test and control groups at p<0.0001; data are also provided in Tables 3 and 4.

Next, we were interested in determining whether specific subsets of SINEs were important in SC formation in other male mammals. Thus, we conducted an initial set of subcloning experiments in the male rhesus macaque (*Macaca mulatta*), examining seminiferous tubules and using similar methodology to that described above. Subclones were sequenced by ABI sequencing and aligned against the UCSC rhesus macaque genome assembly (MGSC Merged 1.0/rheMac2, Jan. 2006) and analyzed by RepeatMasker [13,14]. In total, we analyzed 46.6 Kb from 194 test subclones and 7.3 Kb from 35 control (mock pull-down) subclones (Table 3; Figure 2a). The small set of control subclones were further analyzed and confirmed that the sequences were consistent with genomic averages (Table 3; Figure 2a). Similar to the results from male mice, SINEs were the only DNA category over-represented in the test group, accounting for 16.6% of bp in the test but only 8.1% of bp in controls (p<0.0001; Figure 2a). Subsequent analyses of the individual SINE families demonstrated that the effect was primarily attributable to one class of SINEs; i.e., AluY elements, although AluS elements were also elevated (Table 4; Figure 2b). Thus, it appears that SINEs and, in particular AluYs, serve as binding sites for axial element proteins during the formation of the synaptonemal complex in rhesus males.

**Table 3:** Distribution of different categories of DNA based on sequence analysis of subclones from test (SYCP3) and control (no antibody) ChIP experiments of male macaque

**DNA type**	**Test no. of base pairs (%)**	**Control no. of base pairs (%)**	**Macaque genome ave. (%)**
**SINEs**	7729 (16.6)	595 (8.1)	13.1
**LINEs**	8147 (17.5)	1366 (18.6)	20.4
**LTRs**	3866 (8.3)	678 (9.2)	8.3
**DNA Elements**	864 (1.9)	97 (1.3)	3.0
**Unclassified/other repeats**	0 (0.0)	0 (0.0)	0.2
**Unique sequence**	26040 (55.8)	4624 (62.8)	55.0
**Total Bp Sequenced**	46646 (100.0)	7360 (100.0)	100.0

**Table 4:** **Distribution of different categories of *Alu *repeats from test (SYCP3) ChIP experiments of male macaque**

**DNA type**	**Test no. of base pairs (%)**	**Macaque genome ave. (%)**
***Alu*Y**	1793 (3.8)	1.4
***AluS***	3048 (6.5)	6.6
***AluJ***	931 (2.0)	2.7
***Alu *(total)**	5772 (12.4)	10.7
**Total Bp sequenced** 46646		

## Discussion

Taken together, these results indicate that the youngest and most active SINE subfamilies are intertwined with the establishment of the mammalian SC. However, because of the complex nature of the meiotic prophase axis, we cannot be certain that SINEs complex exclusively with SYCP3. That is, it is thought that the mature axis is comprised of several components: an underlying scaffold, possibly similar to that present in mitotic cells [15], multiple complexes of cohesin proteins that link sister chromatids and possibly homologs [16], and meiosis-specific proteins such as SYCP3 that constitute the axial/lateral elements proper. Since chromosomal loops are evident along the length of the underlying scaffold even in the absence of cohesin and axial/lateral element proteins [17,18], it is possible that SINEs localize to the base of the loops regardless of the presence of SYCP3. Nevertheless, while we cannot be certain of the specific mechanism by which SINES interact with the SC, our results provide evidence of a new meiotic function for repetitive elements. From studies of multiple organisms, it is clear that repetitive sequences are important in the earliest events of meiotic prophase; e.g., telomeric repeats facilitate homolog interactions by formation of the meiotic bouquet [19-27], and studies of C. elegans [28] and Drosophila [29], among others, implicate other specific repetitive elements in the pairing process. However, little has been known of the possible contribution of specific DNA sequences to the formation of the synaptonemal complex in mammals. Our results suggest that a specific category of interspersed repetitive elements plays a role in linking DNA to the axial elements of the mammalian SC. These results extend previous suggestions that rodent axial elements might be bound by various types of repeats [11,12], and indicate that primate males may operate similarly. Further, there may be an evolutionary advantage to this arrangement. SINE elements are dependent upon long interspersed elements for retrotransposition and have evolved in tandem. SINE-LINEs have been some of the most successful elements, both in terms of their numbers within genomes and their ubiquitous presence across lineages. Virtually all mammals have been shown to have active LINE-SINE retrotransposons and although long hypothesized, no requisite biological function has been discovered to counterbalance the mainly disrupt effects of retrotransposition and prevent SINE extinction [30]. It is intriguing to hypothesize that mammalian SINEs may have been initially sequestered and silenced in the SC protein matrix, eventually becoming a requisite component for proper SC formation and meiosis. Given that we observe enrichments for only the most recent active SINE families, the ongoing evolution of SINEs may be intertwined with the evolution of the mammalian meiotic machinery.

## Conclusions

Because B1 and AluY elements are the most actively retrotransposing SINEs in mice and rhesus monkeys, respectively, our observations imply that they may serve a dual function in axial element binding; i.e., as the anchoring point for the SC but possibly also as a suppressor/regulator of retrotransposition.

## Methods

### Sample acquisition

C57BL/6J inbred mice were maintained in a pathogen-free breeding colony at Washington State University (WSU). Protocols for the care and use of the animals were approved by the WSU Animal Care and Use Committee and were in accordance with the National Institute of Health’s standards established by the Guidelines for the Care and Use of Experimental Animals. All procedures were approved by the WSU Institutional Review Board. Testes from sexually mature rhesus macaques (*Macaca mulatta*) were obtained from animals assigned to surgery or necropsy for other purposes, and housed at the California National Primate Research Center (CNPRC) at the University of California, Davis. The CNPRC is fully accredited by the Association for Assessment and Accreditation of Laboratory Animal Care (AAALAC).

### Chromatin immunoprecipitation (ChIP) and whole genome amplification (WGA)

ChIP assays were conducted utilizing seminiferous tubules extracted from one C57BL/6J male mouse and four *Macaca mulatta* males (one testis each for test and control pull-downs), and using the USB ChIP Assay Kit; SYCP3 antibody was obtained from Santa Cruz Biotechnology (Cat. SC-33195). WGA was performed on immunoprecipitated DNA that was first purified utilizing USB’s PrepEase® DNA Clean-Up Kit, with the subsequent amplification performed using the GenomePlex® Complete (WGA2) Kit.

### Subcloning and sequencing

WGA-derived DNA from test and control ChIP experiments was subcloned into pGEM®-T Easy Vectors using standard recombinant DNA techniques. Recombinant plasmids were transformed into JM109 high efficiency competent cells and plasmid DNA isolated using the Wizard® SV 96 Plasmid DNA Purification System. The plasmid inserts were sequenced using BigDye® Terminator v3.1 Cycle Sequencing and were run on a 3730 DNA analyzer. The 454 GS FLX Titanium chemistry platform was employed to generate in-depth DNA sequence coverage of SYCP3 and control WGA-derived DNA from test and control pull-downs. GS 20 library construction and sequencing were performed following standard protocols [31,32] with one major modification; WGA-derived DNA was not sheared but was directly sequenced.

### Sequence analysis

Mouse and macaque subclone insert sequence reads greater than 50 bp in length were aligned to reference genomes (mm9 or rheMac2) with BLAT [33] to determine their best placement, representing the position of the alignment with the greatest sequence similarity (at least 98%) and length (at least 90% of the read). In the case of sequences with multiple best placements, a single best placement was randomly chosen. The repetitive content within each best placement was extracted from the underlying reference genome RepeatMasker annotation.

454 GS FLX trimmed reads greater than 25 bp were treated as above except the best placement sequence similarity had to be at least 95%. The repetitive content within each best placement was extracted from the underlying reference genome RepeatMasker output (mm9) and assigned to the read. The assigned repeat content of the reads was then tallied and tabulated at both the level of individual repeat and repeat family using custom Perl scripts (Tables 1–4). Standard chi-square analyses were used to determine any over-representation of interspersed repeats between the test sequences and expectation.

## Competing interests

The authors declare that they have no competing interests.

## Authors’ contributions

MJ and TH designed and coordinated the study and wrote the manuscript. CV provided the macaque tissue. RR and SS assisted MJ with experimental protocols and JB provided bioinformatics expertise. All authors read and approved the final manuscript.
